# The Effect of the Temperature and Moisture to the Permeation Properties of PEO-Based Membranes for Carbon-Dioxide Separation

**DOI:** 10.3390/polym13132053

**Published:** 2021-06-23

**Authors:** Dragutin Nedeljkovic

**Affiliations:** Department of Chemical Engineering, College of Engineering and Technology, American University of the Middle East, Egaila 15453, Kuwait; Dragutin.Nedeljkovic@aum.edu.kw; Tel.: +956-22251400 (ext. 2196)

**Keywords:** polymeric membrane, carbon-dioxide separation, pollution treatment, zeolite, poly(ethylene oxide), mixed matrix membrane

## Abstract

An increased demand for energy in recent decades has caused an increase in the emissions of combustion products, among which carbon-dioxide is the most harmful. As carbon-dioxide induces negative environmental effects, like global warming and the greenhouse effect, a decrease of the carbon-dioxide emission has emerged as one of the most urgent tasks in engineering. In this work, the possibility for the application of the polymer-based, dense, mixed matrix membranes for flue gas treatment was tested. The task was to test a potential decrease in the permeability and selectivity of a mixed-matrix membrane in the presence of moisture and at elevated temperature. Membranes are based on two different poly(ethylene oxide)-based polymers filled with two different zeolite powders (ITR and IWS). An additive of detergent type was added to improve the contact properties between the zeolite and polymer matrix. The measurements were performed at three different temperatures (30, 60, and 90 °C) under wet conditions, with partial pressure of the water equal to the vapor pressure of the water at the given temperature. The permeability of carbon-dioxide, hydrogen, nitrogen, and oxygen was measured, and the selectivity of the carbon-dioxide versus other gases was determined. Obtained results have shown that an increase of temperature and partial pressure of the vapor slightly increase both the selectivity and permeability of the synthesized membranes. It was also shown that the addition of the zeolite powder increases the permeability of carbon-dioxide while maintaining the selectivity, compared to hydrogen, oxygen, and nitrogen.

## 1. Introduction

The rapid development and globalization since the middle of the 20th has been accompanied with a huge increase in the demand for energy. Although significant efforts and improvements were observed in the field of renewable energy sources (wind, solar, wave etc.), accompanied by the improved safety of nuclear plants, currently, a majority of the energy demand is supplied by the combustion of the fossil fuels. At the current level of industrial development, renewable sources still cannot provide a sufficient and reliable amount of the energy. As carbon-dioxide is one of the main products of the combustion process, huge amounts of it are emitted in the Earth’s atmosphere, causing negative consequences like the greenhouse effect, global warming, or acid rains. Although industry and power plants are the main sources of carbon-dioxide emissions, significant amounts of it are emitted as the consequence of everyday life [1,2]. Therefore, a reduction in the emission of carbon-dioxide has emerged as one of the main challenges for the scientific and engineering community, not only for environmental engineers, but also for chemical or mechanical engineers, physicists, economists, and biologists. As the amount of carbon-dioxide produced is determined by the stoichiometry of the combustion reaction and the efficiency of the combustion process, the main point of interest in regard to emission reduction is the separation of carbon-dioxide from the flue gases. Most constituents of the flue gases (nitrogen, residual oxygen, water vapor) are harmless, so the main goal is to remove the carbon-dioxide from other gases [3]. Current large-scale procedures are accompanied by different disadvantages. Currently, the most common process is based on cryogenics. Flue gases are cooled down in this process and the separation is based on the phase change. The main disadvantage of the cryogenic process is that it requires huge amounts of energy. All constituents of the flue gases have relatively low boiling points at standard conditions, so the cooling of the hot gases is required, and the pressure must be carefully monitored. Part of the heat from the combustion products can be recuperated through the heat exchangers, but this would further increase the complexity of the whole process, making it even more expensive and harder to control. The chemical adsorption is based on the chemical reaction between the (acidic) CO_2_ gas and the alkaline solutions [4]. The use of this process is hindered by the price of the equipment and chemicals. Another disadvantage of this process is the formation of toxic and health-hazardous products [5]. As an innovative approach to this problem, the application of membrane technology for separation has begun to develop in recent years [6]. An advantage of membrane separation of the CO_2_ is that a similar system could be applied not only for the flue gas treatment, but also in enriching the hydrogen produced by the water gas shift reaction [7,8]. Research in the modeling of membrane systems for carbon-dioxide/hydrogen separation in hydrogen production was conducted as well [9]. A suitable membrane for the purpose of separation should have high permeability for carbon-dioxide, and low permeability for all other common constituents of flue gases [10]. Standard porous membranes are not applicable for this purpose as the separation should be performed on the molecular level, which is significantly lower than standard size of the membrane pores [11]. Another problem directly related to the size exclusion process is the size of the carbon-dioxide molecule. This molecule is significantly bulkier than other flue gas molecules (nitrogen, hydrogen, oxygen), which additionally prevents the synthesis of the suitable porous membrane [12]. An alternative approach to this problem would be the application of the dense, non-porous membrane [13]. The gases are dissolved in the membrane material, effectively forming the solid solution, and dissolved molecules are diffusing through the membrane material. The separation is based on different solubility and diffusivities of the different compounds [14]. By this model, components with higher solubility and diffusivity penetrate the permeate side, and the feed (retentate) side remains rich in components with lower values for diffusivity and solubility [15,16].

A suitable material should be mechanically stable to support itself, durable under exploitation conditions of elevated temperatures and pressures, and should maintain separation properties over an extended period of time and over a number of working cycles. According to previous reports, polymers containing ethylene-oxide units in repeating unit can be used for application in carbon-dioxide separation as ethylene-oxide improves the solubility of the carbon-dioxide, maintaining the solubility of the nitrogen and oxygen at the lower levels [17,18]. The main disadvantage of pure poly(ethylene oxide) (PEO) is a high affinity towards the crystallization, which negatively affects permeation properties of the membrane. So, a copolymer that contains PEO units as a co-monomer would be a good choice for this application [19]. This type of the polymer composite may be used either as a self-standing membrane or as an active film coated on the suitable carrier.

As has been observed in previous works, PEBAX 1657 and Polyactive polymers have shown promising results in the field of carbon-dioxide separation [20,21]. PEBAX is a series of commercial polymers produced and supplied by Arkema. It is the thermoplastic elastomer that is poly(amide-b-ether) in structure [17]. A polyamide block is mainly composed of either nylon-6 or nylon-12, and its task is to provide the mechanical stiffness to the membrane material, while a polyether block consists of PEO or tetramethylene oxide and provides the material with good diffusive properties [22,23]. Mechanical and diffusive properties can be fine-tuned by alternating the lengths or ratio of those two blocks [24,25,26]. Polyactive commercial polymer is produced and supplied by IsoTis Orthobiologic (Irvine, CA, USA). It is poly(ethyleneglycol)-co-poly(buthyleneterephthalate). The average molar mass of this polymer is 1500 g/mol with 77 mass% of PEG. An additional advantage of polymers with PEO units is their compatibility with zeolite powder with a high fraction of silicon, which makes them perfect candidates for the polymer matrix for mixed matrix membranes [27].

Due to its smaller size and absence of polarity, it is expected that the hydrogen molecule will have a significantly higher diffusion coefficient in comparison with the relatively massive and bulky carbon-dioxide molecule [28]. As the main purpose of this membrane application is in gas treatments for separation of the carbon-dioxide from the mixture of gases, conditions like the temperature and pressure will be the same for all the constituents of the mixture [29]. Having this in mind, the main mechanism of the action of the polymeric membrane material is to enhance the solubility of the carbon-dioxide while keeping the solubility of hydrogen at minimum [30]. The difference in diffusivity of nitrogen and oxygen is not so prominent as the difference in the sizes of molecules of nitrogen (or oxygen) and carbon dioxide is significantly smaller than in the case of hydrogen and carbon-dioxide. Anyway, their solubility should be kept as low as possible in order to provide good separation [31].

In order to further increase the solubility of carbon-dioxide, various zeolite powders were dispersed in the bulk of the membrane. It was supposed that zeolites, as alumosilicate minerals with different frameworks, would increase the solubility of the carbon-dioxide [32]. The predicted mechanism of action was that the carbon-dioxide molecules would be accommodated in the openings of the framework of the zeolite particles [33]. The zeolite powder should be evenly and homogenously dispersed in the bulk of the membrane. To fulfill this condition, zeolite powder should be dispersible (must not form the aggregates) in the same solvent as the polymer. Particles of the zeolite powder must be in good contact with polymer chains that form the matrix of the membrane, without any void between them. To provide good contact, homogenization additives may be added [34]. The ideal membrane should be transparent or opaque, smooth, without visible inhomogeneity, and without any pin holes. Membranes that fulfilled those conditions were tested for the permeability and selectivity of carbon-dioxide versus other gases under wet and dry conditions. The main question this work tries to answer is whether the elevated temperature and the presence of moisture would decrease the permeability and selectivity of the membrane.

## 2. Materials and Methods

**Figure 1:**
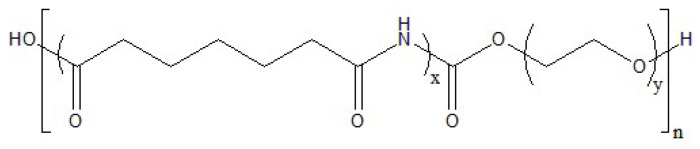
Structure of the PEBAX polymer used for the experiment. Indexes x and y represent numbers of repeating units of nylon-6 and polyethylene oxide respectively; n represents number of repeating blocks.

**Figure 2:**
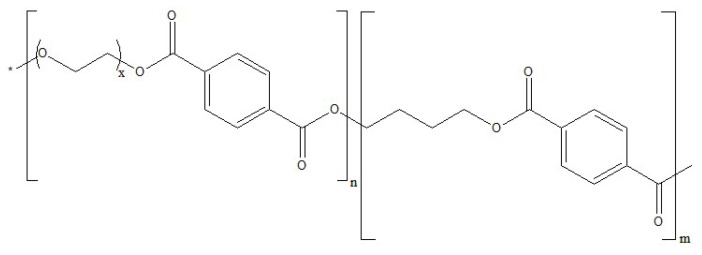
Structure of the Polyctive polymer used for the experiment. Structure of the PEBAX polymer used for the experiment. Indexes m and n represent numbers of repeating units of co-monomers; index x represents number of repeating PEO units; * represents end group.

In this paper, the permeation and selective properties of two different polymers with two different zeolite powders as additives were tested at elevated temperature under wet conditions. The aim of this work was to test if the elevated temperature and presence of moisture would cause a decrease in the permeability and selectivity of the membranes. As a test, the permeability measurements were performed under dry conditions as well. Both of the polymers were used as received and the characterization performed by their respective suppliers. The structures of the polymers are presented on Figure 1 (PEBAX) and Figure 2 (Polyactive).

Alumosilicate powders (zeolites) were dispersed in the polymer matrix in order to improve the permeability of the carbon-dioxide. The permeation properties and the contribution to the overall solubility and diffusivity of zeolites can be described on two different levels. The main property on the higher (macro or bulk) level is surface area per unit mass, which usually determines the zeolite’s capability for adsorption. Zeolite powders used in this research have typical values of area per unit of about 700–900 m^2^/g. On the other hand, on the framework (micro) level, main properties of the zeolite are the orientation of the pore and the diameter of the maximum sphere that can diffuse through it. The orientation of the pores may be in one, two, or three dimensions. Based on the previous research, zeolites with three-dimensional pores were chosen for the investigation as they have shown superior properties in comparison with one- or two-dimensional pores. The zeolites are designed as ITR and IWS with a maximum diameter of the diffusive sphere of 57 pm and 67 pm, respectively, and pore sizes of 64 pm and 82 pm, respectively. The zeolite powders were supplied by NanoScape (Martinsried, Germany) and characterization was performed by the supplier. A relatively high value for the sphere that can diffuse through the framework should provide good solubility and diffusivity of the carbon-dioxide molecule. In analogous experiments with the carbon dioxide, oxygen, and nitrogen under dry conditions, those zeolites showed good compatibility with the chosen polymers. Smooth and homogenous membranes with good permeability and selectivity under the dry conditions were synthesized, so it was supposed that the analogous system would yield good results for the experiments conducted under wet conditions [21]. Both of the selected zeolite powders contain three dimensional pores which have shown better results in comparison with one- or two-dimensional pore powders [21]. ITR and IWS zeolite powders were used due to their high content of silica, which increases the solubility of the CO_2_ [35]. Powders with a high solubility of CO_2_ act as a molecular sieve with diameters of the diffusive sphere sufficiently big to allow the passage of bulky CO_2_ molecule. Once molecules are inside the pore, they are accommodated and diffuse to the permeate side, driven by the difference in pressure between retention and permeate sides.

As the membrane mainly consists of hydrophobic polymer chains and the zeolite particles are electrically charged, an additive which would provide good contact between the particles and matrix is necessary. In previous experiments, it was observed that an improvement in the contact between zeolite and polymer was obtained by the addition of n-tetradecane trimethyl ammonium bromide (n-C14-TMABr). The mechanism of the additive action is that a long, hydrophobic aliphatic “tail” will get dispersed in the bulk of the polymer matrix, while the highly charged “head” with the polar trimethyl ammonium–bromine bond will act as an “anchor” for the zeolite particle. As the additive is compatible with both polymer matrix and zeolite, it should provide a good and homogenous membrane without voids on the contact surface. The additive was supplied by Sigma Aldrich (St. Louis, MO, USA) and used as received.

The membrane preparation procedure was as follows:

PEBAX was dissolved in the mixture of water and methanol (70:30 by mass), at the temperature of 80 °C under reflux. The Polyactive was dissolved in water at the room temperature. Selected zeolite was dissolved in the same solvent as the polymer. The additive was added to the same solution and the solution was mixed by the ultrasound mixer (90 W power, 40 kHz frequency). This solution was added to the solution of the polymer and stirring continued overnight at the same temperature as the pristine polymer. The mass fraction of the zeolite versus polymer was 20 mass% and the fraction of additive versus zeolite was 8 mass%. On the following day, a viscous solution of the polymer, zeolite, and additive was casted on the Teflon surface bordered by the Teflon ring, covered by the non-woven textile and dried in a working laboratory hood overnight. The drying process was relatively slow and no underpressure was applied in order to prevent the formation of bubbles of solvent vapors inside the membrane. One membrane was made of each of the pristine polymers without any additives or fillers in order to test their permeation properties. The formed mixed-matrix polymer membrane was placed on the vacuum line in order to remove any traces of the residual solvents. Dried membranes were used for the dry set of the measurements. The membranes for the wet measurement were prepared by positioning in the closed chamber with liquid water. Then, the chamber was kept overnight at designed temperature and atmospheric pressure, so the liquid–vapor equilibrium was achieved, the membrane could be soaked by the water, and the effects of the water vapor on the permeation properties could be tested.

Permeability was determined and calculated by the time lag method that considers both solubility and diffusivity [36]. The required values were calculated by the following equations [37,38,39]:

(1) $$\alpha_{A/B}=\frac{P_{A}}{P_{B}}=\frac{D_{A}S_{A}}{D_{B}S_{B}}$$

(2) $$D=\frac{l^{2}}{6\theta }$$

(3) $$P=DS=\frac{V_{p}l\left(p_{p_{2}}-p_{p_{1}}\right)}{ART\Delta t\left(p_{f}-\frac{p_{p_{2}}\;+\;p_{p_{1}}}{2}\right)}\times 10^{-10}$$

where: α_*A*/*B*_—selectivity of the membrane for the gas *A* versus gas *B* (ratio of permeability of *A* versus permeability of *B*). *P_A_*—Permeability of the gas *A* in Barrer. *D_A_*—diffusivity of the gas *A* in cm^2^/s. *S_A_*—solubility of the gas *A* in mol·cm^3^/mmHg. *l*—thickness of the membrane in cm. *θ*—time lag in *s*. *V_p_*—permeate volume in cm^3^. *A*—surface area of the membrane in cm^2^. *R*—universal gas constant (62,364 mmHg·cm^3^/mol·K). *T*—temperature in *K*. Δ*t*—time required for the pressure on the permeate side to increase from *p_p_*_1_ to *p_p_*_2_ (Δ*t* is in s; all pressures are in mmHg).

**Figure 3:**
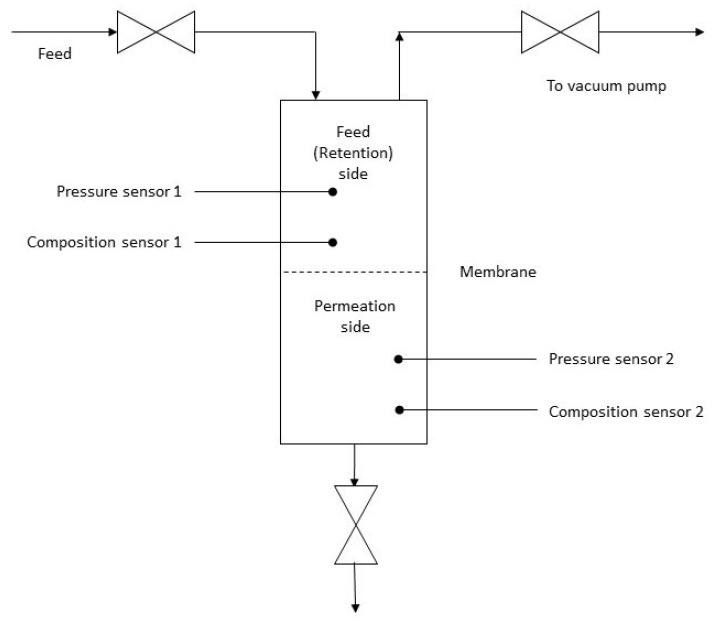
Scheme of the apparatus for the permeability of the dry membrane.

Dry measurements were performed at room temperature and the wet measurements at 30, 60, and 90 °C. The membrane was placed in the measurement chamber and supported by steel mesh. The gas was applied on the feed side with the vacuum on the permeate side. Pressure difference between two side served as the driving force for the diffusion. For the dry measurement, gas was applied directly to the feed side. For the wet measurements, the gas was heated to the desired temperature, bubbled through the water, and applied at the feed side of the membrane. Gases were measured in sequence, and between two different gases, high vacuum was applied on the membrane in order to remove potential residuals of the previously measured gas. The amount of the carbon dioxide on both permeate and retention sides was assessed by the device manufactured and supplied by ABB (model 6515, Zürich, Switzerland) which is a common device for measurements of the composition of flue gases. The thickness of the membrane was measured by Semiconsoft device, model MPROBE40-MSP (Southborough, MA, USA). The apparatus used for the dry measurements is presented in Figure 3.

**Figure 4:**
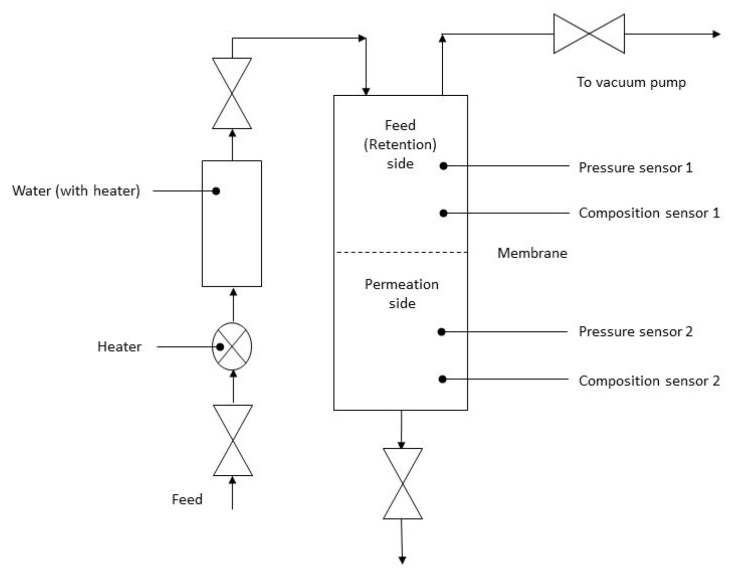
Scheme of the apparatus for the permeability of the wet membrane.

A similar setup was used for the permeability of the wet gases. The gases were heated and bubbled through the distilled water at the appropriate temperature. The apparatus is presented in Figure 4.

Before measuring the permeability of hydrogen, nitrogen, oxygen, and carbon dioxide (in that order), the permeability of helium was measured as well. Helium was chosen as an indicator of the presence of any pin hole or defect on the membrane, as it is a chemically stable, inflammable gas with small, perfectly rounded atoms. The presence of the pin hole was detected by the absence of the time lag and by a sudden increase in the pressure on the permeate side. Membranes with pin holes were discarded, and the new membrane with the same composition was synthesized. The permeability of each gas was measured twice for each membrane, and for the control. The results presented are the arithmetic mean of these measurements. The sequence of the gases was chosen in order to prevent the formation of potentially flammable or explosive mixtures. From the obtained permeability data, the selectivity of each gas was calculated versus carbon-dioxide.

## 3. Results

Six different samples were prepared for the measurements. Two samples (one with each polymer, samples *P_B_* and *P_A_*) were prepared from the pure polymer without the additives, and their permeability and selectivity were measured. The permeability of all membranes is presented in the unit Barrer, which is a common unit in the membrane technology community. The conversion factor between the Barrer and appropriate SI unit is:

(4) $$1\;\mathrm{Barrer}=3.35\cdot 10^{-16}\frac{m^{3}}{m^{2}\cdot \mathrm{Pa}\cdot s\cdot m}$$

Physically, the permeation of 1 Barrer is the permeation of 1 cm^3^ of oxygen through the membrane of the surface area of 1 cm^2^ and the thickness of 1 cm during 1 s, driven by the pressure difference of 1 mmHg, multiplied with factor 10^−10^. The composition of the membranes is presented in Table 1.

**Figure 5:**
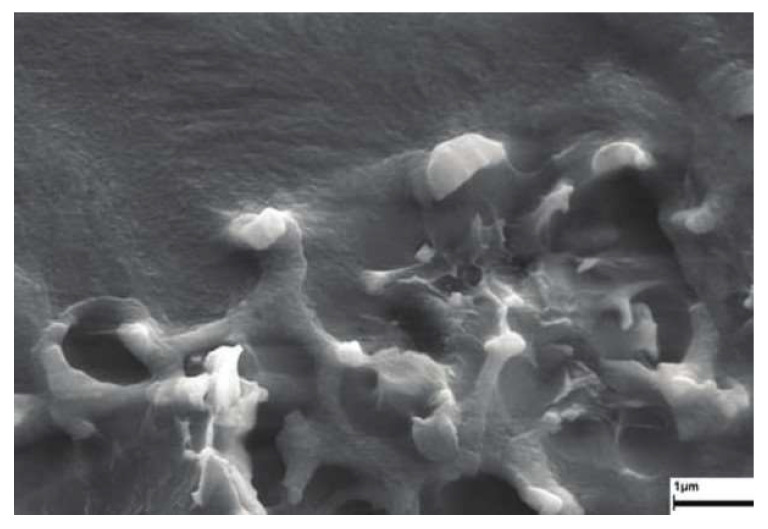
SEM scan of the sample PBW.

The preliminary evaluation was performed by bare eye observation. All synthesized membranes were transparent or slightly opaque, without visible inhomogeneity in appearance and a smooth surface. This indicates that the dispersion of the powder in the membrane matrix was achieved and that the formation of the agglomerates was prevented. To check for potential presence of agglomerates and voids, scanning electron microscopy (SEM) was performed. The SEM photo of the sample PBW is presented in Figure 5.

As can be seen in Figure 5, zeolite powder (lighter particles) is well dispersed in the polymer matrix (gray bulk). The contact between the particles and the bulk is good, as no voids can be observed between the dispersed phase and the matrix. Other samples have shown similar appearance regarding the absence of voids and agglomerates.

The first measurement was performed on all membranes at 30 °C (room temperature) under dry conditions. Measurements were performed in order to obtain the basic permeability data which will be compared with wet measurements. Obtained results for the permeability of all measured gases and selectivity are presented in Table 2.

As can be seen in Table 2, the permeability of all membranes is in the range of expected values (120 Barrer for CO_2_ and 12 Barrer for H_2_) and in good accordance with similar systems obtained in analogous experiments that were conducted previously [21]. As the experiment under dry conditions was successful, measurements at three different temperatures under wet conditions were performed. Results of the measurements at 30 °C under wet conditions are presented in Table 3.

Analyzing the data from Table 3, the composition of the membrane does not play significant role in terms of permeability or selectivity. Obtained results for the permeability of all the gases are within narrow margin of the results. A possible explanation for this may be that both the ITS and IWS zeolites are of the similar chemical compositions, with similar pore orientation. Besides, the membranes made with zeolite additives are similar in composition. Comparing the data from Table 2 and Table 3, it can be seen that the presence of moisture does not significantly change permeability or selectivity. The permeability of CO_2_ is slightly increased, while any change in the permeability of other gasses (if any) is below the error margins of the measurement device. A possible explanation for the influence of the water on carbon-dioxide only (although very slight) is an interaction between CO_2_ and H_2_O on the molecular level. As the solubility of CO_2_ in water is higher than the solubility of H_2_, O_2_, or N_2_ at the same pressure, this effect might contribute to a slight increase of carbon-dioxide permeability. As the permeability of each gas remained the same or slightly increased, no significant changes in selectivity were observed. To test the influence of the elevated temperature on the permeability and selectivity of the membranes, two additional measurements were performed at the temperatures of at 60 °C and 90 °C. Results are presented in Table 4 and Table 5, respectively.

Comparing the permeability data from Table 4 and Table 5, it can be seen that the permeabilities of PEBAX-based membranes are slightly higher than the those of Polyactive membranes. Although the difference is minor, it is present in each sample. This small but consistent discrepancy is in accordance with previous experiments performed with dry samples [21]. A similar relation is observed at 30 °C, for both dry and wet conditions. Thus, it can be concluded that the moisture and elevated temperature influence all polymer–zeolite combinations in the same way. Comparing the data for each of the membranes at different temperatures, it can be seen that the values are very close, often within the range of experimental error. Based on that, it can be concluded that the presence of the moisture and elevated temperature will not significantly change the permeation properties of the membranes, based both on PEBAX or Polyactive, with the additive of either ITS or IWS zeolite powder. At every temperature, the best results for the CO_2_ permeability were obtained for the membranes with ITR as a filler (PBT and PWT). Permeability measurements of the samples based on IWS (PBW and PAW) are slightly below PBT and PAT, but still better than the results of the pristine polymer. This order of values for the permeability is observed for every temperature measured. Moreover, a slight increase in carbon-dioxide permeability is observed with the increase of temperature for every membrane synthesized. The permeability for hydrogen and oxygen increased as well, but with a lower increase which ultimately led to increased selectivity of the membrane. The temperature and the partial pressure of water played no role in the selectivity of nitrogen versus carbon-dioxide as the differences were minor (if any) and can even be attributed to the experimental error. Based on the obtained results, it is reasonable to conclude that polymers with PEO blocks filled with zeolite powder are promising materials for the future development of the membranes for carbon-dioxide separation. After the measurements, the membranes were inspected for any visible decay or defect due to the exposure to moisture and increased temperature, but no damage was observed. However, the behavior of the polymer material under repeated measurements and cycles of increasing and decreasing temperature and pressure will be investigated in further experiments.

## 4. Discussion

In this paper, the behavior of mixed matrix membranes for carbon-dioxide separation at elevated temperatures and in the presence of moisture was tested. The main task was to test if the presence of the moisture at high temperature would degrade the membrane and decrease permeability or selectivity. The targeted application of this type of polymeric membrane is flue gas treatment and the decrement of carbon-dioxide emission. Two different polymers (PEBAX 1657 and Polyactive) and two different zeolite powders (ITR and IWS) were tested in the presence of n-tetradecane trimethyl ammonium bromide (n-C14-TMABr) as additive. The measurements were performed at three different temperatures with saturated water vapor pressure at given temperature. Obtained results indicate that the increase of the temperature (thus, the partial pressure of water vapor) slightly increases the permeability of all gases. However, the permeability of the carbon-dioxide is increased at a higher rate in comparison to hydrogen, oxygen, and nitrogen, which ultimately led to a slight increase in the selectivity of carbon-dioxide versus other gases. The addition of both of zeolite powders slightly increases the permeability of carbon-dioxide. Permeabilities of other gases remain the same, which at the end slightly increases selectivity (CO_2_ versus H_2_, O_2_ and N_2_). The addition of ITS powder increases the permeability of carbon-dioxide for 4.2–5.8% at the same temperature for the PEBAX membranes. The permeability of hydrogen or oxygen remains unchanged by the addition of ITS powder. Although a slight increase is present at every temperature, such change never exceeded 1.5%, ultimately yielding an increase of selectivity by 5–6%. Zeolite powders had slightly greater influence on the carbon-dioxide permeability of Polyactive based membranes with the increase ranging between 5.0% and 8.3%. The change of permeability of the other three gases was in the same range as in the case of PEBAX-based membranes, with the change in permeability not exceeding 1.5%, and the overall increase in selectivity was 6–7%. A possible explanation for this is the high affinity of carbon-dioxide to both of the zeolite powders used for this experiment (both IWS and ITS are zeolites with high content of silica). ITS powder has shown slightly higher permeability and selectivity in comparison to IWS at all temperatures. As pore openings of ITS are smaller than openings of IWS, it may be concluded that the pores of ITS serve as sieves on the molecular level, where CO_2_ molecule can be accommodated, hindering the potential diffusion of smaller molecules. On the other hand, bigger pore openings of IWS still leave sufficient space for smaller molecules to pass, even if carbon-dioxide molecule is accommodated in it.

The presence of moisture and increased temperature slightly increased the permeability of carbon-dioxide and its selectivity versus other gasses. Comparing the PEBAX based samples at different temperatures, it can be seen that a temperature increase from 30 °C to 90 °C, together with the presence of moisture, increases the permeability of CO_2_. Differences in permeability for other gases were between 1.5% and 2% (Overall increase in selectivity was about 4%). Elevated temperature and moisture had similar effects on the permeability and selectivity of Polyactive-based membranes. An increase in the permeability of carbon-dioxide was 6–8% and the increase for other gases was about 1.5% (increase in selectivity was about 5%). A probable reason for this observation is the higher solubility (thus, stronger intermolecular forces) of CO_2_ in water in comparison with H_2_, N_2_, and O_2_ at the same pressure.

As the differences in permeability were minimal, a *t*-test was performed in order to check the statistical significance of measured results. Calculated *p*-values for PEBAX-based membranes showed statistical significance for pristine polymer (sample PB; *p* = 0.040) and for the sample with ITS zeolite (sample PBT, *p* = 0.038), while the statistical significance is not observed for IWS membrane (sample PWT, *p* = 0.058). Similar results were calculated for the samples based on Polyactive. Statistical significance was observed in the measurements of the pristine polymer (sample PA *p* = 0.030) and sample with ITS powder (sample PAT, *p* = 0.044), and it was not observed for the sample containing IWS (sample PAW, *p* = 0.060). However, those evaluations were performed on a sample of only two measurements. As one of the future plans is to test the behavior of membranes under repeated measurements cycles, a bigger sample for the statistical significance tests will be available.

As the main task of this work was to test if an increase in temperature and the presence of moisture would decrease the permeation properties and selectivity of the membrane, it can be stated that this type of polymeric membrane appears to be a good starting point for further investigations. Potential directions for further research include the assessment of the behavior of the polymer in repeated cycles of measurement and an investigation of the possible deterioration of the properties. Other research topics in this field would include the measurement of permeation properties at even higher temperatures (closer to the real exploitation in the combustion processes) and further decreases in the thickness of the membrane.

## Figures and Tables

**Table 1:** Composition of the membranes used for the dry and wet measurements of the permeability and selectivity.

Sample	Polymer	Zeolite	Additive	Thickness, µm
PB	PEBAX	-	-	207
PBT	PEBAX	ITR	TMAB	188
PBW	PEBAX	IWS	TMAB	197
PA	Polyactive	-	-	220
PAT	Polyactive	ITR	TMAB	235
PAW	Polyactive	IWS	TMAB	230

Samples are labeled as follows: PXY; X represents type of polymer for matrix (B for PEBAX, A for Polyactive); Y represents type of zeolite (T for ITR, W for IWS, no letter for samples made of pristine polymer). TMAB stand for of n-tetradecane trimethyl ammonium bromide.

**Table 2:** Permeability and selectivity of samples measured at 30 °C under dry conditions.

Sample	P(CO_2_), Barrer	P(H_2_), Barrer	P(O_2_), Barrer	P(N_2_), Barrer	α (CO_2_/H_2_)	α (CO_2_/O_2_)	α (CO_2_/N_2_)
PB	118	13.7	5.2	2.1	8.6	22.5	55
PBT	123	13.6	5.2	2	9	23.7	62
PBW	119	12.9	4.9	2	9.2	24	60
PA	110	12.5	5.4	1.8	8.8	20.5	60
PAT	120	14	5.9	2	8.6	20.3	58
PAW	118	13.1	5.8	2	9	20.3	60

**Table 3:** Permeability and selectivity of samples measured at 30 °C under wet conditions.

Sample	P(CO_2_), Barrer	P(H_2_), Barrer	P(O_2_), Barrer	P(N_2_), Barrer	α (CO_2_/H_2_)	α (CO_2_/O_2_)	α (CO_2_/N_2_)
PB	120	13.7	5.2	2.1	8.8	22.7	56
PBT	127	13.9	5.3	2	9.1	23.7	63
PBW	118	12.9	4.9	2	9.4	24.1	60
PA	115	12.8	5.5	1.9	9	20.8	61
PAT	123	14	6	2.1	8.8	20.3	60
PAW	120	13.2	5.9	2	9.1	20.4	60

**Table 4:** Permeability and selectivity of samples measured at 60 °C under wet conditions.

Sample	P(CO_2_), Barrer	P(H_2_), Barrer	P(O_2_), Barrer	P(N_2_), Barrer	α (CO_2_/H_2_)	α (CO_2_/O_2_)	α (CO_2_/N_2_)
PB	121	13.8	5.3	2.1	8.9	23	56.5
PBT	130	14.3	5.4	2.1	9.1	23.9	63
PBW	122	13	5	2	9.4	24.3	61
PA	117	12.9	5.6	1.9	9.1	21	61
PAT	125	14.2	6.1	2.1	8.8	20.5	61
PAW	121	13.2	5.9	2	9.2	20.6	62

**Table 5:** Permeability and selectivity of samples measured at 90 °C under wet conditions.

Sample	P(CO_2_), Barrer	P(H_2_), Barrer	P(O_2_), Barrer	P(N_2_), Barrer	α (CO_2_/H_2_)	α (CO_2_/O_2_)	α (CO_2_/N_2_)
PB	125	14	5.4	2.2	8.9	23	57
PBT	133	14.3	5.5	2.1	9.3	24	64
PBW	123	13	5.1	2	9.5	24.3	61
PA	121	13.3	5.7	2	9.1	21.3	62
PAT	126	14.2	6.1	2.1	8.9	20.6	61
PAW	124	13.3	6	2.1	9.3	21	61

## Data Availability

Not applicable.
